# Elucidation of Therapeutic Peptide Binding Partners from Isolated Mitochondria

**DOI:** 10.7759/cureus.2898

**Published:** 2018-06-30

**Authors:** Ali Mozayan, Annette Khaled

**Affiliations:** 1 College of Medicine, University of Central Florida, Orlando, USA; 2 Burnett School of Biomedical Sciences, University of Central Florida, Orlando, USA

**Keywords:** ct20p, bax, breast adenocarcinoma, mitochondria, therapeutic peptides

## Abstract

CT20p is a protein derived from the C-terminus of Bax. It has selective cytotoxicity for cancer cells, such as the sensitive triple-negative MDA-MB-231 breast adenocarcinoma cells, but not normal cells like the resistant MCF-10A epithelial breast cells. To understand the reason for the peptide’s selective toxicity, a "pull-down" experiment with biotinylated CT20p (biotin-CT20p) and whole-cell protein lysates from breast cancer and normal cells were performed. These studies revealed that CT20p binds to a cytosolic protein called chaperonin-containing TCP-1 (CCT), a molecular chaperone that folds actin and tubulin. However, this method could not detect possible rare interactions made by CT20p with mitochondrial proteins. To determine whether CT20p is associated with mitochondrial proteins as part of the mechanism by which it induces cell death, mitochondrial protein lysates from MDA-MB-231 and MCF-10A cells were isolated and a streptavidin-agarose pulldown procedure using biotin-CT20p was performed. Protein interactions were visualized by sodium dodecyl sulfate polyacrylamide gel electrophoresis (SDS-PAGE) using silver staining. The results of the experimental procedure showed that biotin-CT20p did not "pull down" any observable mitochondrial proteins from the sensitive MDA-MB-231 cells, indicating that the peptide may not interact with mitochondrial proteins in breast cancer cells. Rather, the interactions observed with biotin-CT20p were with mitochondrial proteins derived from resistant MCF-10A cells, indicating that these interactions were not driving the cancer-selective cell death process. The absence of CT20p-associated proteins from the mitochondrial lysates of MDA-MB-231 breast cancer cells supports the hypothesis that CT20p, unlike the parent protein, Bax, exerts its cytotoxic effects via a cytosolic protein.

## Introduction

Therapeutic peptides are a rapidly growing class of drugs that can be used to treat a wide array of diseases and disorders, including cardiovascular disease, infectious disease, and cancer. Peptides offer greater potency and less non-specific effects than chemically synthesized drugs. There is a need to identify new peptides with cytotoxic activity that could have therapeutic value. One way to discover new peptides is to derive these from proteins with known cytotoxic action that target and inhibit specific organelles or proteins within cells. For example, many therapeutic peptides in pre-clinical or clinical use resemble naturally occurring antimicrobial peptides that can perturb membranes. Of these, peptides that target mitochondria and facilitate the release of intramembrane contents, leading to cell death, could have clinical value. Mitochondria serve key functions in both sustaining life, through energy production by oxidative phosphorylation, and in death, by mediating cytochrome C release and apoptosis.

A number of inhibitory peptides that bind to anti-apoptotic components have been developed and tested, for example, in the treatment of cancer. Oblimersen, one such inhibitor that binds to Bcl-2 messenger ribonucleic acid (RNA), was believed to show promise in reducing Bcl-2 levels but failed to demonstrate sufficient efficacy in Phase III clinical trials. Other drugs have attempted to directly inhibit the interactions between Bcl-2 protein family members. The most successful of these thus far has been ABT-199, a modification of navitoclax, in an attempt to reduce its prevalent side effect of thrombocytopenia [1-2]. However, a major gap in the literature is the general lack of mechanistic understanding concerning how therapeutic peptides act at the organelle level to promote intracellular changes that, in turn, alter cell physiology and viability [3]. To address this gap, the recently developed cytotoxic peptide, CT20p, could be used to define target molecules and mediators that drive peptide-induced cell death. This information could help develop a companion diagnostic approach to improve the clinical application of CT20p. Thus, the objective of this project was to identify mitochondrial proteins that interact with CT20p, which could serve as biomarkers to screen cancer patients for a therapeutic benefit.

CT20p is a derivative of the C-terminus of the pro-apoptotic protein Bax, and is an example of a synthetic peptide with possible pore-forming characteristics that display mitochondrial and cytoskeletal effects [4-6]. However, despite its origins, CT20p does not mirror the parent protein Bax in terms of function. For example, CT20p increases mitochondrial membrane potential and triggers the fusion and clustering of mitochondria, whereas Bax depolarizes mitochondria and triggers mitochondrial fission. In addition, CT20p inhibits key cytoskeleton components, leading to a loss of migration, cell detachment, and the death of human cancer cells but not normal cells. Indeed, CT20p selectively targeted and killed the human triple negative breast cancer cell line MDA-MB-231 but not the normal breast epithelial MCF-10A cells. In contrast, the overexpression of Bax in normal cells would result in cell death. This suggests that CT20p may interface with intracellular proteins in a unique way distinct from Bax. Thus, the hypothesis investigated was that CT20p binds to cancer-specific mitochondrial proteins in order to promote mitochondrial-associated changes that contribute to cancer cell death.

## Materials and methods

Breast adenocarcinoma cells (MDA-MB-231) were cultured in Dulbecco’s modified Eagle medium. The normal breast epithelial cells (MCF-10A) were cultured in mammary epithelial cell growth media.

Mitochondrial lysates from MCF-10A and MDA-MB-231 breast adenocarcinoma cells were prepared by utilizing the Thermo Fischer Mitochondrial Isolation Kit (Waltham, Massachusetts, United States) and a dounce tissue grinder for mechanical douncing.

Five hundred µg of mitochondrial lysate from MCF-10A and MDA-MB-231 cells were mixed with 10 µg of biotin or biotin associated with the CT-20 peptide. The mixture was then placed into a rotating incubator for three hours at room temperature. Afterward, 20 µL Thermo Scientific Pierce Agarose beads (Waltham, Massachusetts, United States) coated with streptavidin were added. The mixture was then left overnight at 4 °C in a rotating incubator.

The next step was centrifuging the mixture at 10,000 rpm for 10 minutes to collect the beads. The supernatant was drained off and the beads were subject to a 500 µL wash consisting of 25 mM Tris, 150 mM NaCl, and 0.1% NP40. Three washes were completed, and between each wash, the beads were centrifuged at 10,000 rpm for 10 minutes.

Twenty-five µL of Laemmli sample buffer was then added and the mixture was centrifuged at 13,000 rpm for 10 minutes. The mixture was placed on a sodium dodecyl sulfate-polyacrylamide gel electrophoresis (SDS-PAGE) gel and electrophoresis was performed.

After completing the gel electrophoresis, the gel was silver-stained using the Thermo Scientific Pierce silver stain (Waltham, Massachusetts, United States) for mass spectrometry following manufacturer instructions.

The stained SDS-PAGE was examined for the presence of protein bands. The molecular weights of the bands were estimated by comparing them to the SeeBlue Pre-Stained Standard marker (Thermo Fisher Scientific, Waltham, Massachusetts, United States). No statistical methods were utilized in this experiment.

## Results

**Figure 1:**
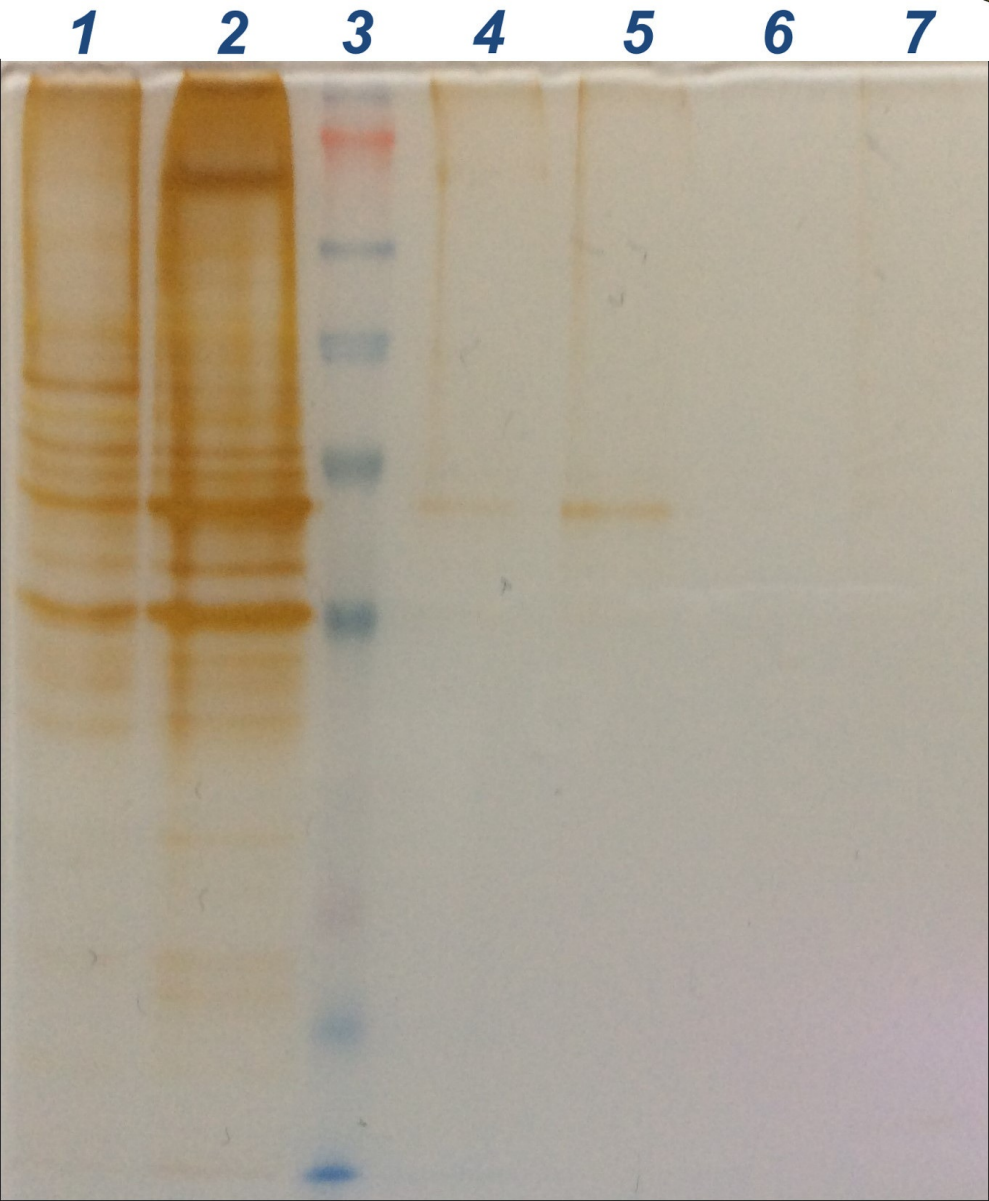
SDS-PAGE gel electrophoresis Lanes from left to right: (1) MDA-MB-231 mitochondrial lysate; (2) MCF-10A mitochondrial lysate; (3) SeeBlue Pre-Stained Standard (marker); (4) Biotin MCF-10A pulldown; (5) Biotin+CT20p MCF-10A pulldown; (6) Biotin MDA-MB-231 pulldown; (7) Biotin+CT20p MDA-MB-231 pulldown SDS-PAGE: sodium dodecyl sulfate-polyacrylamide gel electrophoresis

A silver-stained SDS-PAGE gel resulting from the completion of the experimental procedure is shown in Figure 1. The protein band in the biotin+CT20p MCF-10A pulldown lane is representative of a molecular weight of approximately 50 kDa. The gel does not reveal any protein bands in the lanes corresponding to MDA-MB-231 pulldowns. The experimental procedure was completed twice with the other stained gel obtained showing a similar banding pattern.

## Discussion

The absence of pulled-down protein from MDA-MB-231 mitochondrial lysates adds evidence to the concept that CT20p does not exert its effects via direct interaction with mitochondrial proteins. Instead, CT20p binds to mitochondrial proteins from normal MCF-10A cells. It is likely that this interaction is not a driver of cytotoxicity given that normal breast epithelial cells are resistant to the killing action of the peptide. A possible avenue of further study would be to excise the protein band from the biotin+CT20p MCF-10A pulldown and identify it using mass spectrometry.

Other parallel studies recently performed by the Khaled lab suggest that the mechanism of cytotoxicity with CT20p may be through interactions with the cytosolic protein CCT (chaperonin-containing TCP1). A similar pulldown procedure was carried out using whole-cell lysates from MDA-MB-231 and MCF-10A cells, which showed interactions between CT20p and CCT subunit proteins. Further, CCT overexpression induced by transfection with the CCT2 gene enhanced susceptibility to CT20p’s cytotoxic effects.

## Conclusions

The pulldown procedure employed in this experiment was designed to capture stable protein-protein interactions between CT20p and mitochondrial components. It remains possible that protein interactions between CT20p and mitochondrial components may be transient or rare events not stable enough to be isolated with this procedure. A gel-free system for direct mass spectrometry identification of protein "pull-downs" could be done. In addition, it is possible that CT20p exerts cytotoxic effects via a pore-forming mechanism or through protein-lipid mitochondrial interaction, which would not be captured using a streptavidin-agarose pulldown.
